# Understanding the Attitudes of Clinicians and Patients Toward a Self-Management eHealth Tool for Atrial Fibrillation: Qualitative Study

**DOI:** 10.2196/15492

**Published:** 2020-09-17

**Authors:** Boon Piang Cher, Gayatri Kembhavi, Kai Yee Toh, Jananie Audimulam, Wei-Yan Aloysius Chia, Hubertus JM Vrijhoef, Yee Wei Lim, Toon Wei Lim

**Affiliations:** 1 Centre for Health Services and Policy Research Saw Swee Hock School of Public Health, National University of Singapore National University Health System Singapore Singapore; 2 Department of Family Medicine and Chronic Care, Department of Patient and Care University Hospital Maastricht, Maastricht, The Netherlands Panaxea Amsterdam Netherlands; 3 Saw Swee Hock School of Public Health Yong Loo Lin School of Medicine National University of Singapore Singapore Singapore; 4 Department of Cardiology National University Heart Centre National University Hospital Singapore Singapore

**Keywords:** mHealth, qualitative research, atrial fibrillation, self-management, chronic disease, mobile phone

## Abstract

**Background:**

Atrial fibrillation (AF) is the most common heart rhythm disorder and poses a growing disease burden worldwide because of an aging population. A multidisciplinary approach with an emphasis on patient education and self-management has been demonstrated to improve outcomes for AF through the engagement of patients in their own care. Although electronic tools (e-tools) such as *apps* have been proposed to provide patient education and facilitate self-management, there have been few studies to guide the development of these tools for patients with AF.

**Objective:**

This study aims to explore the perceptions of patients and health care providers (HCPs) and their attitudes toward the use of e-tools for the self-management of AF. It also seeks to elicit the factors that contribute to these attitudes.

**Methods:**

Semistructured qualitative interviews with HCPs and patients were conducted to understand the interpretations and expectations of an e-tool that would be used for the self-management of AF. Interview data were analyzed using an exploratory thematic analysis approach to uncover emergent themes and infer ideas of preferred features in a device. A modified technology acceptance model was developed as a framework to help interpret these findings. Data from the HCPs and patients were compared and contrasted.

**Results:**

Both patients and HCPs thought that an e-tool would be useful in the self-management of AF. Although both groups favored educational content and monitoring of blood pressure, patients expressed more passivity toward self-care and an ambivalence toward the use of technology to monitor their medical condition. This appears to be related to factors such as a patient’s age, social support, and their attitudes toward technology. Instead, they favored using the app to contact their HCPs.

**Conclusions:**

This study provides insights into significant differences in the attitudes of patients and HCPs toward the use of e-tools for self-care against their priorities. Understanding patients’ motivations and their needs are key to ensuring higher acceptance of such tools.

## Introduction

### Background

Atrial fibrillation (AF) is the most common and clinically significant arrhythmia. It is an important risk factor for serious adverse events such as stroke, heart failure, and early mortality. Worldwide, there were an estimated 11 million cases of AF in 2013, which was underestimated because of the high prevalence of asymptomatic AF [1,2]. Its prevalence increases with age by 5% to 15% and is expected to rise 2.5-fold in the next 50 years. A recent study revealed that hospitalizations for AF increased by 420% from 767 to 3986 per 1 million Korean population from 2006 to 2015 [3]. The overall cost of AF in the same study showed an increase from EUR 68.4 million (US $86.2 million) to EUR 388.4 million (US $431.1 million) in the same period, highlighting the additional health care and economic burden from the condition [3].

In addition to providing AF care, the European Society of Cardiology guidelines underlined the importance of patient involvement in the self-management of AF [4]. The guidelines further state that patient “education is a prerequisite for informed, involved patients and patient-centred care.” Nevertheless, overall patient knowledge about AF remains poor [5-8]. In recent years, electronic tools (e-tools) have been used as platforms for patient education and disease self-management. Some e-tools have shown to improve patient outcomes by either improving disease knowledge or medication adherence monitoring [9,10].

There remains a paucity of such app-based tools developed for patients with AF [11-13]. Therefore, the aim of this study was to determine perceptions of health care providers (HCPs) and patients and their attitudes toward an e-tool known as Self-management and Education Tool for AF patients (SETAF) that can be used to improve AF knowledge and self-manage the condition at home. The factors that affect how patients’ HCPs respond to the e-tool and the functions and features they would consider desirable were also studied. Insights from this study may aid the further development and implementation of SETAF to a larger audience.

### Theoretical Framework

The technology acceptance model (TAM) described by Davis [14] is one of the most commonly used models to predict the acceptance of technology. According to the TAM, technology acceptability is dependent on a user’s perceived usefulness and the perceived ease of using the device. Perceived usefulness was defined as the tendency to use an app depending on a user’s belief that it will enhance their task performance. Meanwhile, perceived ease of use is the user’s belief that a particular system is easy to use. The combination of the 2 perceptions determined each user’s attitude, behavioral intention (BI) to use, and ultimately, the actual use of the system. In the field of health care, the TAM was modified to include constructs from other health-related models [15]. Holden and Karsh [15] noted that as TAM was not designed “specifically in or for health care context,” the use of TAM in its generic form “may not capture or indeed may contradict some of the unique contextual features of computerised health care delivery.” The model also does not consider social factors that potentially influence a user’s decision to use technology [16]. In a study that explored women’s acceptance of seeking health information through models, the construct on self-efficacy from the social cognitive theory of Bandura was included and was found to be highly correlated to BI [17]. Another study examining the use of smartphones for chronic disease management also extended the TAM model to include constructs from the health belief model and other social and demographic factors [16]. Some of these additional constructs were found to have an influence on technology acceptance.

## Methods

### Study Design

This exploratory study used qualitative semistructured interviews to understand the perceptions of HCPs and patients with AF and their attitudes toward using the AF self-management e-tool.

### Study Population

Purposive sampling was conducted to gather insights from HCPs who (1) were currently working in the outpatient cardiology clinic of a tertiary university hospital in Singapore and (2) have extensive experience working with patients with AF. We approached 23 cardiologists, nurses, and pharmacists who were working in the heart clinic through email to participate in the study. In total, 12 HCPs (4 physicians, 4 nurses, and 4 pharmacists) agreed to participate and were interviewed between February and April 2016.

A total of 16 patients with AF and their caregivers were recruited from the same cardiology clinic. The inclusion criteria were (1) age >21 years, (2) ability to speak English, and (3) hospitalizations in the past 6 months. Patients who had a history of cognitive impairment or were otherwise unable to provide informed consent for the study were excluded. Patients and their caregivers were interviewed together if they were both present at the clinic. The rationale for this was that most patients with AF are elderly and often rely on their caregivers as support to use e-tools. These caregivers either facilitate the patients’ access to these tools or may in fact use them on the patient’s behalf. Hence, patient and caregiver dyads were interviewed together, as the presence of the caregiver may affect how patients interacted with the e-tool. A total of 11 patients and patient and caregiver pairs agreed to participate in the study and were interviewed in July 2016. Patients who declined to be interviewed cited reasons such as needing to leave the hospital after their appointment or were uninterested in technology.

### Interview Procedure

All HCPs and patients were interviewed either in the offices of the HCPs or in a quiet room within the clinic. Semistructured interviews were conducted using an interview guide (Multimedia Appendix 1). The questions sought to understand the current care provided by the cardiology clinic, patients’ experiences with self-care, the potential for the use of e-tools in AF self-management, preferred type of e-tool, and preferred features of the e-tool. Each interview lasted between 30 and 60 min.

### Materials

Most patients with AF in Singapore are elderly and have limited formal education. According to data from the Singapore Department of Statistics, more than 63% of the population aged 65 years and older have only had formal education below the secondary school level [18]. Therefore, the demonstration tablet provided a visualization form. During the interview, patients were given tablets installed with a self-care program app (demonstration version) and a blood pressure (BP) machine loaned by Koninklijke Philips N.V. This is an Android tablet app built within the Philips Motiva Platform and has a touchscreen-based interface. Features of the e-tools were introduced to participants during the interview, and they were asked to provide their perspective about these features. The demonstration version consisted of 2 main functions. First, it provides information and educational content for patients to learn about AF and its management. This is in the form of videos related to general health, health-related reminders and messages, and survey questions. Second, the tablet also has monitoring functions and is linked wirelessly to the BP machine. This allows it to automatically log BP and heart rate of the patients, which is then uploaded to the Motiva cloud-based database.

### Data Analysis

All 24 interviews were digitally recorded and transcribed verbatim by a professional transcriber. Most of the interviews were conducted in a colloquial form of English widely spoken in Singapore, known as Singlish, and the transcripts as well as the quotes in this manuscript retain the nonstandard grammar used. The transcripts were analyzed using thematic analysis using ATLAS.ti version 8 (ATLAS.ti Scientific Software Development GmbH) to organize the data. An initial codebook was developed by 2 researchers (BC and GK) using 3 transcripts. The remainder of the interviews were coded by one researcher (BC), with reliability checks performed on 7 interviews (GK). First-order codes were identified, then subsequently grouped into second-order nodes and, finally, key themes. The analysis was performed for each participant group separately (HCPs and patients) and then examined for thematic connections between the participant groups.

### Ethics Approval

This study was approved by the domain-specific institutional review board (DSRB 2015/00940). Researchers conducting the interviews explained the purpose of the study and clarified any questions from the participants. Participants were informed that their data would be anonymized and that their participation in the study was voluntary, before signing informed consent documents. Patients were provided with SGD 10 (US $6.90) reimbursement for their participation.

## Results

### Participants Profile

The characteristics of the study participants are summarized in Table 1. HCP participants had worked in a cardiology clinic of a tertiary hospital for an average of 8 years (range 2-13 years). The patients’ ages ranged from 50 to 76 years, and they had a diagnosis of AF between 0.5 and 17 years. There were mostly male and Chinese patients, and nearly all were on warfarin therapy.

**Table 1:** Health care providers’ and patients’ characteristics.

Characteristics			Values
**Health care providers (n=12), n (%)**			
	Doctors		4 (33)
	Nurses		4 (33)
	Pharmacists		4 (33)
Years in practice of the health care providers, mean (range)			8 (2-13)
**Patients (n=11)**			
	Patients interviewed alone, n (%)		7 (64)
	Patients interviewed with caregiver, n (%)		4 (36)
	Age (years), mean		61
	Male, n (%)		7 (64)
	**Ethnicity, n (%)**		
		Chinese	8 (73)
		Malay	2 (18)
		Indian	1 (9)
	**Anticoagulation, n (%)**		
		Warfarin	9 (82)
		Direct oral anticoagulant	2 (18)

### Themes

**Figure 1:**
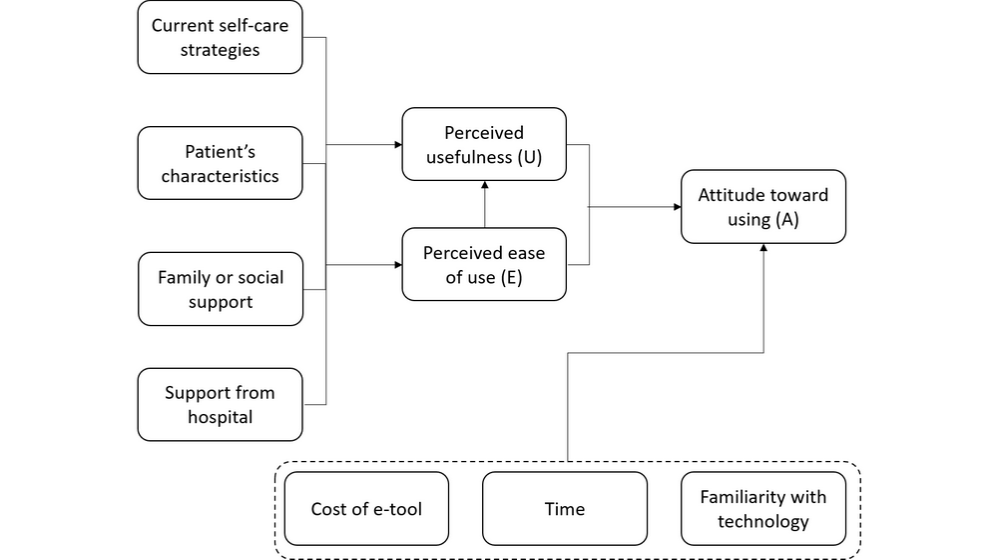
Proposed modified technology acceptance model. A: attitude toward use; E-tool: electronic tool; E: perceived ease of use; U: perceived usefulness.

A modified TAM framework (Figure 1) was developed using the findings from this study for 2 reasons: (1) the modified model sought to highlight the social factors that influence perceptions and acceptability and (2) the TAM construct of *actual use of the system* had to be removed, as this was an exploratory study with no actual utilization data. The modified TAM framework illustrates the major themes of this study. The analysis focused on how various factors influenced participants’ perception of the usefulness of an e-tool and their attitudes toward using such a tool. The thematic analysis combines the findings from the HCPs and the patients. The results include comparisons between participant groups, where appropriate.

The themes derived from our interviews with patients and HCPs are summarized in Table 2 and presented in more detail below.

**Table 2:** Summary of themes from patient and health care provider interviews.

Themes	Patients	Health care providers	Other observations
Current self-care strategies	Reading material about diet Recording diet and medications	Advised to monitor BP^a^, diet, and exercise Provided reading material	Patients did not follow advice on exercise and diet
Patient characteristics and attitudes toward self-care	Negative attitude generally, did not feel they can make a difference	Younger patients more educated and more likely to self-care	N/A^b^
Family and social support	Family support important enabler	Family support important enabler	N/A
Support from hospital	Access to health care advice important Reliant on health care providers for health information	Language barrier affects patients’ ability to understand their condition	N/A
Perceived usefulness	e-tool^c^ can be useful for self-management Valued BP monitoring, educational videos, and support from health care providers	Valued patient support groups, reminders about diet, medication, or clinic appointments	Discrepancy between what patients and health care providers valued
Technical preferences for e-tool	Preferred smartphone based Some preferred larger screens	Integration with eHealth record important	Both groups emphasized the accuracy of monitoring tools and multilanguage support
Attitudes toward using e-tool	Mostly reluctant to use as unfamiliar with technology Concerned about lack of time and cost of devices	More receptive than patients Can empower patients and provide reassurance Concerned that patients may be resistant to using the tools Lack human touch	N/A
Redefining the use of non–e-tools	Generally preferred paper-based tools for education and recording BP measurements	Engage primary and community care to customize care	N/A

^a^BP: blood pressure.

^b^N/A: not applicable.

^c^e-tool: electronic tool.

#### Current Self-Care Strategies

In addition to general consultation for AF, HCPs mentioned that the clinic also provided patients with self-care strategies. Dietary consultation featured prominently for patients who were prescribed warfarin (a blood thinner to reduce stroke risk) because of the large number of food-drug interactions. Patients confirmed the provision of a booklet for foods they should avoid consuming. HCPs also advised patients to regularly attend clinical consultations; instructed them to measure and record their BP and heart rate at home; and advised them to exercise regularly and make other lifestyle changes, such as lowering stress levels.

Some self-care strategies were adopted by patients who mentioned that they went for frequent checkups, watched their diet, and consumed medications as directed, for example, through the use of pillboxes. However, in direct contrast to what was discussed by the HCPs, most patients mentioned that they did not exercise or measure their BP regularly because they were not advised to do so by clinicians:

Interviewer: So, did the doctor advise you that you should monitor your heart rate at home?

Patient: No, he never ask me to do that.

I: What about blood pressure?

P: No, no.

Patient 9

From the HCPs’ standpoint, medication and dietary restriction adherence can be challenging for some patients. This is particularly true for patients on warfarin, as dosage can be frequently altered, and it may be difficult for patients to keep up with the changes. The HCPs were also concerned about potential drug-drug interactions because of polypharmacy from other conditions and interactions with food that may contain traditional Chinese medicine ingredients. Finally, patients often cited a lack of time or comorbidities as a reason for not exercising regularly.

#### Patient Characteristics and Attitudes Toward Self-Care

Patient characteristics largely defined their attitudes toward self-care. The HCPs noted that younger patients tend to be more educated and have a higher health literacy level and thus were better able to care for themselves. Better self-care was also observed in patients who had higher levels of motivation and technology-savviness. For example, one patient mentioned that he read medical books to better understand AF and experimented with different diets:

I start looking at the medical books like science guy you know so...ok, this is AF you know... ventricle [is this], not this, ok, so in us... there is some... misbehaviour somewhere in... those things and then that’s how suddenly, you know, bad thing come up, you see? So what are what are precautions, I must do and so on, you see? So I, I...I [have] been very careful!

Patient 8

Conversely, negative attitudes in patients were seen as a major barrier to self-care. Some participants mentioned that apart from diet and medications, they did not make other lifestyle changes because they *could not be bothered* or were *too lazy* to do so. Others felt useless and hopeless, and these patients believed that they could not do much to improve or manage their condition:

I: Do you have experience using blood pressure machine?

P: I have [it] at home but I don’t bother with this. I kept there quite some time already.

I: Why not? What is it that is preventing you from using it.

P: I [am too] lazy to go and take (it) out [to use].

Patient 4

#### Family and Social Support

High levels of family involvement or good social support were facilitators of self-care. For some patients, caregivers helped them to either monitor their diet and/or medications and were also involved in educating them about their condition.

Conversely, lack of support from family members was a barrier to self-care. For example, dietary restrictions were noted by some patients and HCPs to be challenging. For these patients, family members who were not used to cooking with or eating healthier food options (eg, healthier oils and whole grains) were initially resistant to making dietary changes:

The oil must change. Now we never take the vegetable oil at home, we must buy the soya bean oil, the olive oil for him. At first a bit difficult, because my children can’t take it when I cook with that oil. They say it’s a bit different, the taste is a bit different. Then I said it’s good for your father, must try to [change] the oil.

Caregiver 7

#### Support From Hospital

Patients with AF who are prescribed warfarin require regular monitoring of blood coagulation levels. The HCPs indicated that low-income patients who had difficulty attending frequent follow-up appointments at the clinic were loaned the international normalized ratio (INR) testing machines to self-test at home. Different levels of telecare were described by the HCPs. Telecare was provided to these patients by pharmacists from the anticoagulation clinic calling them to monitor their INR levels and to advise them on titrating their warfarin dosages:

[The] patient can do a phone consult. That means [the] patient comes, then [has the] blood [test], then they will go. Then pharmacy will trace the blood [results] later, because waiting for the blood test and all that right, so it takes some time, so they will let the patient go home so anything “I’ll update you on the dosage.” That means the dosage titration will be done over the phone, then the pharmacist will call the patient or the caregiver, “okay you can continue the drugs until 2 weeks’ time then we see you again,” something like that. There’s another one where a patient can go to a polyclinic, the nearest polyclinic.

Nurse 4

Interestingly, although some patients mentioned that they occasionally discussed issues about their condition with their HCPs over the phone, this was not connected to the INR home monitoring service. They thought that the ability to call nurses when faced with problems was helpful to them and could facilitate self-care. Many patients were also unaware that they could do their blood tests at home. The availability of the INR machine is also an area of some confusion. Although the machine is available for purchase in the hospital pharmacy, one HCP mentioned that it was not currently available for purchase in Singapore.

In terms of health information seeking behavior, the majority of the participants relied extensively on their HCPs for information. This was particularly evident in patients who did not self-monitor their BP, as they felt that it was frequently checked in the hospital. Similarly, these participants only sought information about AF from their HCPs and did not actively search for information or speak to their friends or families about their condition:

I: Besides from the doctor, did you find out [information] from anyone else?

P: No, I don’t!

I: What about from... the internet?

P: No!

I: Then, your friends?

P: No!

I: Or, anyone with the same conditions?

P: I only... want to interact with the doctors, other than that, my friends and all, they don’t know my case.

Patient 11

Despite the patients’ reported reliance on HCPs, the HCPs stated that patients have problems understanding AF, in part because of language barriers between the HCPs and the patients. The language barrier was felt to result in misconceptions about AF in the patient population.

#### Perceived Usefulness

Overall, the majority of participants felt that an e-tool would be useful for self-management. In particular, features that were found to be useful by both HCPs and patients were BP monitoring and logging, educational videos, and support from HCP. In contrast, features such as patient support groups and reminders about diet, medications, and clinic appointments were consistently seen as useful by HCPs but not by patients. The following section outlines the participants’ perceptions of the various suggested features.

#### Educational Videos

HCPs stressed the importance of education in AF self-management. In their view, patients require education about the management of AF (such as pharmacotherapy and other lifestyle advice) and knowing when and where to seek help. Moreover, as some patients may not necessarily know how to navigate the e-tool, education about how to use the tool was also seen to be important. When shown a demonstration of the e-tool, patients found the content about medication and dietary advice useful. Patients also wanted e-tools to include information about financial aid and other conditions.

#### BP Monitoring and Logging

Patients liked the ability to use the e-tool to monitor and automatically log BP results. In addition, both HCPs and patients would like to see this feature extended to other conditions. For example, one suggestion was that the same tool could be used to measure and log blood glucose levels of diabetic patients. Another suggestion was to have BP monitoring on top of existing heart rate monitoring in wearables.

#### Interaction With HCPs

Another feature of an e-tool desired by both HCPs and patients was interaction with a member of the clinical team. The level of interaction could range from having the HCP monitor a patient’s vital signs through the e-tool or having a quick feedback or question section for patients to pose their questions to their HCP, to having a chat-bot and a messaging system with the clinical team. Patients generally preferred methods that provided more interaction with their HCP, as it would resolve issues faster.

#### Reminders for Medications, Diet, and Appointments

The HCPs felt that having a reminder system for medication use, diet, and clinical appointments may help to improve adherence in patients. However, this sentiment was not shared by most patients. Both HCPs and patients felt that it would be useful if the tool could provide a list of current medications prescribed to the patient. The HCPs also suggested that the cost of medications could be included in the tool.

#### Patient Support Group

A virtual patient support group was suggested as a useful element of the e-tool by HCPs. However, most of the patients felt that this was not useful. Reasons included not wanting to be overburdened with reading about other patients’ issues, being misled by false information, feeling that their condition was not serious enough to warrant such a group, and an unwillingness to reveal health information to others:

I doubt people want to [reveal] their condition to us unless it is to [their] doctor. Usually patients I don’t think they will want to let you know what’s their outcome [is]. Unless it’s your family member.

Patient 4

#### Technical Aspects of E-Tools: General Preferences

In general, participants had varied preferences in terms of the e-tool platform. Many participants felt that having an AF app on a smartphone would be more convenient as they always have access to their phones. Others felt that it would be easier to access the content if it were on bigger screens, for example, on a tablet or computer. Apart from apps, HCPs also mentioned the convenience of websites as they can be accessed from computers, tablets, and smartphones:

Computer will be better. Phone is also difficult. Bigger screen better. Tablet ok.

Patient 1

In terms of technical qualities of the e-tool, the HCPs and the patients emphasized the importance of having accurate information and accurate readings (eg, BP) in the e-tool. Both groups also emphasized the importance of making the e-tool a multilanguage tool. Both patients and HCPs also hoped to see an interactive e-tool. Furthermore, the HCPs added that there should be seamless data integration between the e-tool and the hospital system, although some expressed concerns over confidentiality and privacy with a linked system.

#### Attitudes Toward Using E-Tools

In general, HCPs were more receptive to the idea of using e-tools than patients. They believed that the use of e-tools could empower patients, provide them with reassurance, and, in the process, help reduce costs. The potential of using an e-tool to gain easy access to patients’ self-monitoring data was seen to be advantageous.

Patients, in contrast, were less enthusiastic about e-tools. They thought that e-tools were generally useful and convenient, as they would be able to view information instantly. However, most of them expressed an unwillingness to use the tool. The primary reason for their reluctance was largely because of unfamiliarity with technology. Some mentioned that they do not own any of these devices (smartphone and tablet) and they do not know how to use the internet. This uncertainty about technology also extended to the perception that e-tools would be complicated and troublesome. Patients felt that e-tools were impractical and were unwilling to learn how to use one. This perceived difficulty was also coupled with the belief that medical terms are complicated, and thus, the content of the e-tool would be equally difficult to understand:

I: If I was to say that I want to introduce a tool like that to help you measure your heart rate, your blood pressure, all these things, would you find it useful?

P: I, I find it not practical use for me lah. I don’t think so.

I: Why is it not practical? Is there any reason?

P: No reason at all.

Patient 5

Lack of time and costs also contributed to the patients’ unwillingness to use an e-tool. Those who were working long hours prioritized rest over the use of an e-tool. Many felt that the cost of the e-tool would be high and indicated that they were only willing to pay up to SGD 200 (US $138) for the e-tool should it be developed for mass use. They also suggested that the e-tool could be paid for by Medisave (a mandatory savings scheme for health care) or Medifund (a government health care assistance scheme):

But before that I will ask, all these, right, will you charge [to] us? Because my husband is not working, I’m not working myself, [because] my daughter is from special school. Now I only have help from elder son, he [is] the one who support us now, but he himself got ... to pay [the] bill so one household. So I myself go hospital under Medifund, that is why I ask you first because we are... [having difficulty with] our finances also.

Caregiver 7

Despite their more positive attitude toward e-tools, HCPs had some reservations. HCPs worried that it would be difficult to convince patients to rely more on e-tools, as it lacks the *human touch* that patients seek during clinic visits. This is particularly so for patients who have frequent follow-ups. Moreover, HCPs were concerned that their patients’ inability to use technology may hinder the adoption of e-tools. They also noted that an e-tool had the potential to increase anxiety if patients overmonitored themselves. The e-tool would be a constant reminder to patients about their condition and may affect their well-being. E-tools with a messaging function may also be an additional burden to HCPs as they may have to constantly respond to patients’ messages.

#### Redefining the Use of Non–E-Tools

Patients mentioned that they were comfortable with printed material to provide information about their condition. For example, patients preferred the booklet of dietary restrictions that are currently provided to them by the AF clinic as opposed to having this information in an e-tool. Patients felt that they could also record BP measurements in notebooks rather than having the readings sync directly to the e-tools. In addition to pamphlets, HCPs suggested books, posters, roadshows, and the use of educational videos in the clinic to help educate patients:

I: Apart from all this I have shared with you, instead of putting them in electronic platform, I give you in, say, a book, is it better for you?

Caregiver: Ya I think it’s better because I can read it. If I don’t understand, I can ask my son ... I can concentrate what is this [and] what is [that].

Caregiver 7

One interesting suggestion by HCPs was to use primary and community care services to help patients manage their condition closer to home. HCPs believed that general practitioners would have a better understanding of the patient’s preexisting conditions, which would help in care management. However, they were concerned about the current cultural preference among patients of seeing specialists in hospitals for heart conditions and that this culture may be hard to change.

## Discussion

Overall, the results from the interviews indicated that having an e-tool to help patients self-manage AF was acceptable to both clinicians and patients. In particular, both parties thought that having more educational content about AF was useful and that monitoring and logging of vital signs through the e-tool was convenient. Having the app on a smartphone appeared to be the preferred platform, given that the majority of patients had them, although some preferred the bigger screen sizes of tablets and computers.

A noteworthy finding was that there were important differences in the preferences of patients and HCPs. The latter wanted features that would enhance their clinical work, such as the ability to integrate data between the e-tool and the hospital network and quantify AF symptoms remotely. In contrast, patients’ interest in the e-tool centered around access to advice from HCPs, BP monitoring, and education about their condition. Nonetheless, there appears to be significant barriers to patient acceptance of such e-tools, which underscores the need to design such tools with patients’ needs in mind.

### Reluctance Among Elderly Patients to Use E-Tools

Patients’ current self-care behaviors are a strong determinant in defining their attitudes toward using e-tools. These self-care behaviors and strategies are, in turn, determined by individual-level characteristics and facilitated by the level of support from family and friends and support from the wider environment, such as from the hospital. For instance, although the HCPs reported asking patients to monitor their BP, the patients in this study did not consider this to be useful. Coupled with a general disregard for self-care, this meant that they did not consider BP monitoring a routine part of AF management.

Currently, patients’ self-care mechanisms include the use of pillboxes, a booklet on dietary restrictions, and logging BP results in notebooks. However, the majority of the participants had a passive attitude toward self-care. Including these features in the e-tool, although potentially useful, may not translate into incremental benefits for patients who do not see the value of self-monitoring. Even among those who were currently self-monitoring, their unfamiliarity with technology created a perception that the device would be difficult to use, leading to a negative attitude toward using e-tools in general.

From a broader perspective, the prevailing environment does not appear to promote the use of e-tools for self-management. Patients with AF are generally older and have multiple comorbidities in addition to AF, such as hypertension and diabetes. Consequently, the patients in this study made frequent trips to the hospital for the management of other conditions due to fragmented specialty care. Frequent clinic contact perpetuates patients’ reliance on the hospital and the HCPs. Although the HCPs mentioned that the e-tools can help reduce trips to the hospital, this may not be beneficial for the patients who are already used to making regular clinic visits. Furthermore, if the content of the e-tool does not include other chronic conditions, it may not significantly reduce the overall need for hospital visits. To overcome this, a more integrated approach to managing these multiple conditions will be needed both in the form of clinical care as well as in the design of e-tools. A potential approach would be to integrate chronic care clinics for patients with AF [19].

### Comparison With Prior Work

Our findings were largely similar to those of other eHealth studies in Singapore. In designing a lifestyle app for overweight pregnant women, Lau et al [20] found that these women also preferred to use smartphones as they are user-friendly and convenient. The need for multilanguage platforms was also reflected in their study. However, unlike our study population, these women expressed a preference for peer support to provide additional information during their pregnancy. The reason for this is uncertain, but the relatively younger patients in the study may be more comfortable relying on multiple information sources through social networks in a manner akin to social media.

Another study looked at apps to improve medication adherence in oncology patients, who valued educational and behavioral interventions [21]. Older patients and those who were less educated were also unlikely to use such smartphone health apps. In our study, although some patients preferred the use of smartphone apps, we also found that some other patients valued the larger screens of tablets or computers as they make it easier to read.

### Advantages of the Modified TAM Framework

The TAM framework was originally devised to study the factors that contribute to the attitude toward the BI to use new technology [14]. However, this does not include some factors that impact the use of technology in a health care context. Hence, the modified TAM framework was developed to include the effects of external social or clinical factors on how patients interact with e-tools. In this study, we found that a patient’s age, social support, and their attitudes toward technology as well as their self-care had important influences on how they perceived the usefulness and ease of use of e-tools. These psychosocial factors are not included in the original TAM, but, for our patients, could influence whether they use an e-tool. This framework extends the TAM beyond more technical considerations, such as the specification of the e-tool and the user interface, and allows a more complete assessment of how patients may respond to eHealth interventions.

### Implications for AF Care

Specific to self-monitoring tools for patients with AF, there are other published studies that demonstrated that patients were generally satisfied with a mobile self-care and medication adherence app [12,13]. In the study by Hirschey et al [12], the majority of the participants reported using the medication reminder feature, despite stating that they would have remembered to take their medication without the app. Participants also liked that they were able to check their heart rates quickly. This is in stark contrast to our findings that such features in an e-tool were not seen to be useful and illustrate the importance of understanding the patient population for whom an e-tool is designed. In the study by Hirschey et al [12], the patients had an average age of 59 years, and the majority had at least some college education. Our participants were older and had much less formal education; more than 63% of Singaporeans aged 65 years or older in 2018 did not attain more than primary school education [18]. They generally had little or no experience using e-tools, given that care was mostly done within the health care setting. Such patient characteristics were likely to be influential in how patients perceive e-tools and need to be considered when designing them.

It is unsurprising that the HCPs in our study believed that the e-tool would only be useful for those patients who were already engaged in regular self-care for AF management. These patients were likely to be younger, have a higher level of education, have better health literacy, were motivated to care for themselves, and were more likely to use technological tools in their daily lives.

Another key finding of this study was that the content and functions of the e-tools suggested by the HCPs did not address what patients thought would be most useful to them. Poor medication and dietary adherence were some of the main concerns from the HCPs’ perspective, and they felt that having educational content and reminders would help patients better manage their condition. However, these were not the main barriers faced by the patients, as the majority claimed to have no difficulty with adherence. What patients valued was the ability to contact or interact with HCP as they still perceived them to be the most reliable source of information and advice. This is likely a reflection of the heavily hospital-centric model of outpatient specialist care in Singapore where most patients with AF receive their routine health care. As such, patients strongly preferred direct access to their HCPs.

Given patients’ current reliance on frequent hospital visits to access their HCPs, patients may benefit more from improved integration of care between hospitals and primary care settings for their chronic conditions, including AF. A systematic review by Gallagher et al [19] showed that multidisciplinary team and community support for patients with AF improved outcomes such as a reduction in all-cause mortality and cardiovascular-related hospitalizations. This supports the need to shift chronic care management away from the hospital. The combination of a well-designed, user-centric e-tool and right-siting health care delivery into the community may be the key to improving overall patient outcomes and may also deliver cost benefits to the health care system.

### Limitations

This study has some limitations worth discussing. First, although the purpose of showing patients a prototype of the e-tool was to facilitate understanding and guide them in answering questions, the presence of the prototype may have limited their expression of ideas and restricted the conversation to their opinions on the features in the sample tablet as opposed to the generation of possible features for the e-tool. Second, because of the strict inclusion criteria, only English-speaking participants were recruited for the study, as the prototype was only available in English. This resulted in a cohort from a narrow demographic in Singapore and made a meaningful analysis of the influence of demographic factors impossible, especially given the small number of patients involved. Nonetheless, it is likely that our study subjects closely reflected the patients most likely to use such e-tools in the real world. We recognize that non-English speakers may have different perceptions of e-tools. Thus, an exploratory study with non–English-speaking participants is crucial before implementing the e-tool countrywide.

The patient and their caregivers were interviewed together in this study, as elderly patients with AF often rely on their caregivers to access e-tools. However, as these were joint interviews, we were not able to analyze their responses separately, and we cannot comment on whether there were any differences between patients and caregivers. It is possible that they may separately respond differently to the e-tool, but we believe that as they are likely to interact with the e-tool in everyday situations as a dyad, interviewing them as a pair would allow a more realistic understanding of how they respond to the e-tool.

### Conclusions

This study provides insights into the acceptability of e-tools as part of AF self-management from the perspective of both HCPs and patients. Educational content and monitoring ability of the e-tool were seen as useful features in patient self-care, but there was discordance between what HCPs and patients perceived to be most useful. Patients’ passivity toward self-care in general will be a challenge when trying to engage them in the use of e-tools, and understanding the target patient population is crucial in designing a e-tool.

## Appendix

Multimedia Appendix 1 Interview guides for health care providers and patients.

## Abbreviations

AF: atrial fibrillation
BI: behavioral intention
BP: blood pressure
e-tools: electronic tools
HCP: health care provider
INR: international normalized ratio
SETAF: Self-management and Education Tool for AF patients
TAM: technology acceptance model
