# Supplementary material for: Understanding the Attitudes of Clinicians and Patients Toward a Self-Management eHealth Tool for Atrial Fibrillation: Qualitative Study
Source: JMIR Hum Factors. 2020 Sep 17;7(3):e15492. doi: 10.2196/15492 (PMC7530695; doi:10.2196/15492)
Supplement: Multimedia Appendix 1 [file humanfactors_v7i3e15492_app1.docx]

**Appendices**

1. **Topic Guide – healthcare provider interviews**

| **SECTION 1: Introduction** |
| --- |
| - How long have you been working with AF patients? |
| **SECTION 2: Knowledge** |
| - Can you explain what AF is to me? - What happens to a patient after they are diagnosed? |
| **SECTION 3: Existing sources of support and coping mechanisms** |
| - How would you describe your patients’ feelings at the point of diagnosis? - What do you advise your AF patients to do to manage AF? - What support do AF patients receive from the healthcare /hospital side? - Do you know if they have any external help managing AF? - If yes, - From where? - What do you think of this support? |
| **SECTION 4: Perceived barriers** |
| - What do you think about patient self-management in AF? - Could you tell me if there are any specific actions a patient can take to self-manage AF? - What are the difficulties your AF patients might face in the self-management of AF? |
| **SECTION 5: Intervention design** |
| - Are you aware of any electronic tools available to help patients to self-manage AF? - If yes   - What tool?  - Do your patients currently use such a tool?  - What do you like about it?  - What do you dislike about it?   - Earlier, we talked about the difficulties AF patients face with the self-management of AF, in your opinion would electronic tools such as websites, computer-based tools or smartphone/tablets apps be helpful to them? - How would these tools be helpful? - Between the three choices – websites, computer-based tools and smartphone/tablet apps, which do you, think would suit your patient’s needs best? - Could you tell me why? - As a practitioner, are there any specific features would like your patients to have in this tool you just picked? - Do you foresee any problems with using _(insert choice here)_? |
| **SECTION 6: Other** |
| - What do you think about any other tools that could help your patients self-manage AF, besides the ones discussed above? - Out of all the ways to manage AF that we have discussed, what is in your opinion the ideal tool for your patients to use for AF self-management? - Any other comment or ideas? |

1. **Topic Guide - patient interviews**

| **SECTION 1: Introduction** |
| --- |
| - How long have you been an AF patient? |
| **SECTION 2: Knowledge and attitude of AF (AF)** |
| - Had ever you heard about AF before you were diagnosed? - At the point of diagnosis, what did you know about AF? - What is your understanding of AF now? - Can you explain what AF is to me? |
| **SECTION 3: Existing sources of support and coping mechanisms** |
| - How would you describe your feelings when they were diagnosed? - How would you describe your feelings now? - How has life changed for you after AF diagnosis? - How do you manage AF? - What support have you received from the hospital side to manage your AF? - Other than the doctor, have you looked for any other support to manage your AF? - If no - Why not? - If yes - From where? - Why did you look for this support? - What do you think of this support? |
| **SECTION 4: Perceived barriers** |
| - Could you tell me if there are any specific actions that you take to self-manage AF? - What challenges do you face when you try to carry out these actions? |
| **SECTION 5: Intervention design** |
| - Are you aware of any electronic tools to help you manage AF better? - If yes - Do you currently use such a tool? - What do you like about it? - What do you dislike about it? - Considering the challenges you have just shared, would electronic tools such as websites, computer based tools, or smart phone/ tablet apps be helpful to you? - How would these tools be helpful? - Between the three choices – websites, computer-based tools and smartphone/tablet apps, which do you think would suit your needs best? - Could you tell me why? - Are there any specific features you would like to have in it? - Do you foresee any problems with using _ (insert choice here)_? |
| **SECTION 6: Other** |
| - What do you think about other tools that could help you self-manage AF, besides the ones discussed above? - Out of all the ways to manage AF, in your opinion what is the most ideal tool for you to use to manage your AF? - Any other comments or ideas? |

1. **Topic Guide - caregiver interviews**

| **SECTION 1: Introduction** |
| --- |
| - How long have you been a caregiver for patient? |
| **SECTION 2: Knowledge and attitude of AF** |
| - Had ever you heard about AF before your patient was diagnosed? - At the point of diagnosis, what did you know about AF? - What is your understanding of AF now? - Can you explain what AF is to me? |
| **SECTION 3: Existing sources of support and coping mechanisms** |
| - How would you describe your patient’s feelings when they were diagnosed? - How would you describe their feelings now? - How has life changed for them after AF diagnosis? - How do they manage AF? - What tasks do you assist the patient with? - How do you help the patient manage AF? - Other than the doctor, has the patient sought any support to manage the AF? - If yes, from where? - What do you think of this support? |
| **SECTION 4: Perceived barriers** |
| - Do you think the patient can handle AF management by themselves? - What challenges do you face when you help the patient manage AF? |
| **SECTION 5: Intervention design** |
| - Are you aware of any electronic tools to help patient manage AF better? - If yes - What? - Does patient currently use such a tool? - What do they/you like about it? - Considering the challenges you have just shared, would websites, computer based tools, or smart phone/ tablet apps be helpful to the patient? - Considering the challenges you have just shared, would websites, computer based tools, or smart phone/ tablet apps be helpful to **you** when it comes to assisting the patient manage AF? - How would these tools be helpful? - Between the three choices – websites, computer-based tools and smartphone/tablet apps, which do you, think would suit your patient’s needs best? - Could you tell me why? - Are there any specific features your patient would like to have in it? - Do you foresee any problems with using _ (insert patient choice here)_? |
| **SECTION 6: Other** |
| - What do you think about other tools that could help your patient self-manage AF, besides the ones discussed above? - Out of all the ways to manage AF that we have talked about, what is the ideal tool for you to use to manage your AF? - Any other comments or ideas? |
